# The Role of Taste Receptor mTAS1R3 in Chemical Communication of Gametes

**DOI:** 10.3390/ijms21072651

**Published:** 2020-04-10

**Authors:** Michaela Frolikova, Tereza Otcenaskova, Eliska Valasková, Pavla Postlerova, Romana Stopkova, Pavel Stopka, Katerina Komrskova

**Affiliations:** 1 Laboratory of Reproductive Biology, Institute of Biotechnology of the Czech Academy of Sciences, BIOCEV, Prumyslova 595, 252 50 Vestec, Czech Republic; Michaela.Frolikova@ibt.cas.cz (M.F.); tereza.otcenaskova@natur.cuni.cz (T.O.); eliska.valaskova@ibt.cas.cz (E.V.); pavla.postlerova@ibt.cas.cz (P.P.); 2 Department of Zoology, Faculty of Science, Charles University, BIOCEV, Vinicna 7, 128 44 Prague 2, Czech Republic; romana.stopkova@natur.cuni.cz (R.S.); pstopka@natur.cuni.cz (P.S.); 3 Department of Veterinary Sciences, Faculty of Agrobiology, Food and Natural Resources, University of Life Sciences Prague, Kamycka 129, 165 00 Prague 6, Czech Republic

**Keywords:** acrosome reaction, chemotaxis, chemoattractant, gamete, *L-glutamate*, mouse, sperm, TAS1R family, mTAS1R3 receptor

## Abstract

Fertilization is a multiple step process leading to the fusion of female and male gametes and the formation of a zygote. Besides direct gamete membrane interaction via binding receptors localized on both oocyte and sperm surface, fertilization also involves gamete communication via chemical molecules triggering various signaling pathways. This work focuses on a mouse taste receptor, mTAS1R3, encoded by the *Tas1r3* gene, as a potential receptor mediating chemical communication between gametes using the C57BL/6J lab mouse strain. In order to specify the role of mTAS1R3, we aimed to characterize its precise localization in testis and sperm using super resolution microscopy. The testis cryo-section, acrosome-intact sperm released from *cauda epididymis* and sperm which underwent the acrosome reaction (AR) were evaluated. The mTAS1R3 receptor was detected in late spermatids where the acrosome was being formed and in the acrosomal cap of acrosome intact sperm. AR is triggered in mice during sperm maturation in the female reproductive tract and by passing through the egg surroundings such as *cumulus oophorus* cells. This AR onset is independent of the extracellular matrix of the oocyte called *zona pellucida*. After AR, the relocation of mTAS1R3 to the equatorial segment was observed and the receptor remained exposed to the outer surroundings of the female reproductive tract, where its physiological ligand, the amino acid *L-glutamate*, naturally occurs. Therefore, we targeted the possible interaction in vitro between the mTAS1R3 and *L-glutamate* as a part of chemical communication between sperm and egg and used an anti-mTAS1R3-specific antibody to block it. We detected that the acrosome reacted spermatozoa showed a chemotactic response in the presence of *L-glutamate* during and after the AR, and it is likely that mTAS1R3 acted as its mediator.

## 1. Introduction

In mammals, fertilization occurs in the female reproductive tract, and after ejaculation, sperm must overcome several barriers before they reach the egg. Only a small percentage of ejaculated sperm enters the oviduct and it is known that during their journey mammalian sperm use chemotaxis [1]. This is a short-distance guidance mechanism that works only within the order of millimeters [2]. Positive chemotaxis is a directed movement up a concentration gradient of chemical factors, called chemoattractants, which influence receptors on the sperm surface and modulate sperm behavior [2,3,4]. Key physiological sperm maturation processes such as the capacitation, hyperactivation and acrosome reaction are triggered during the passage of sperm through the female reproductive tract and they are potentially sensitive to chemotactic mechanisms [5].

The onset of sperm capacitation happens inside the oviduct and represents a series of physiological and cellular changes which provide sperm with the ability to fertilize an egg. Extensive membrane reorganization, which occurs during capacitation and is initiated by cholesterol efflux, is a prerequisite for the acrosome reaction (AR). AR is a final step of sperm maturation. It is defined as a Ca^2+^ dependent exocytosis of the acrosomal vesicle and only acrosome-reacted sperm are able to bind to and fuse with an egg [6]. Generally, in mammals, a natural inductor of the sperm AR is the *zona pellucida*, which is a gycoprotein surrounding of the egg. However, mouse sperm undergo a so-called spontaneous AR [7,8], which happens before sperm reach the *zona pellucida* site [9] and was also described in sperm of *Tas1r1*^−/−^ mice [10]. The spontaneous AR is probably triggered while sperm pass through the oviduct, specifically the upper isthmus [11,12,13]. Only a low percentage of sperm (5%) that reach the ampulla, a place in the oviduct where fertilization occurs, are acrosome-intact. Despite all the current progress in knowledge of mouse sperm maturation, it still remains unclear whether sperm responsiveness to chemoattractants depends on sperm acrosomal status and whether sperm head could respond to the local presence of chemoattractants prior to or after the acrosome reaction.

Taste receptors (TAS) were firstly described as sensory receptors in taste buds of the lingual epithelium [14]. These receptors are divided into two subfamilies—taste receptor type 1 (TAS1R) [15,16] and taste receptors type 2 (TAS2R) [17,18]. TAS1 receptors are G-protein coupled receptors and they are responsible for sweet and umami taste [19]. In general, there are three monomeric members of the TAS1R subfamily, TAS1R1, TAS1R2 and mTAS1R3; however, they can be activated only if they heterodimerize. The heterodimer TAS1R1 + TAS1R3 forms the umami receptor that senses all L-amino acids in rodents but only *L-glutamate* in humans [20], whereas heterodimer TAS1R2 + TAS1R3 is responsible for detection of sweet tastes. Bitter taste perception is mediated through the TAS2R receptors subfamily, whereas salty and sour tastes are based on ion channels.

Taste receptors are not only present in the oral cavity; they also occur in many other tissues [5]. Importantly, the presence of all three members of the TAS1R subfamily, mTAS1R1, mTAS1R2 and mTAS1R3, were observed in mouse testis and knockout mice proved the importance of these receptors for physiological sperm development [21]. Moreover, all three receptors from the TAS1R subfamily are also detected in mouse epididymal spermatozoa [22]. Interestingly, analyses of a nutrient composition fluid from the reproductive tract of female mice showed the presence of 19 amino-acids, including glutamate, which is the ligand for the mTAS1R1/mTAS1R3 heterodimer [23]. In addition, glutamine and glutamate were two of the five major amino-acids detected in oviductal fluid; however, sperm chemotactic responsiveness to glutamate has not yet been addressed. There are known molecules already described as being responsible for mediating various roles in sperm-specific guiding mechanisms, for example, opsins play roles in thermotaxis [24] and olfactory receptors [25] are suggested to be involved in sperm chemotaxis [26]. Taking all this into account, it is feasible that TAS1R1/TAS1R3 on sperm could serve as receptors in chemotaxis.

This study aimed to answer whether the short-distance chemotaxis of sperm could be mediated via mTAS1R3. The identification of specific compartmental localization of mTAS1R3 in sperm heads relating to the integrity of the acrosome vesicle was preformed using super-resolution microscopy, and its localization after the acrosome reaction was specified. Based on the receptor sperm-head location in, a) intact and b) acrosome-reacted sperm, we targeted sperm behavior in the presence of the mTAS1R3 ligand *L-glutamate*. In addition to the immunofluorescent analysis, this study also compares the mRNA expression level of *Tas1r3* in 10 selected mouse tissues.

## 2. Results

### 2.1. The Analysis of mRNA Expression of Tas1r3 Gene in Mouse Tissue

**Figure 1:**
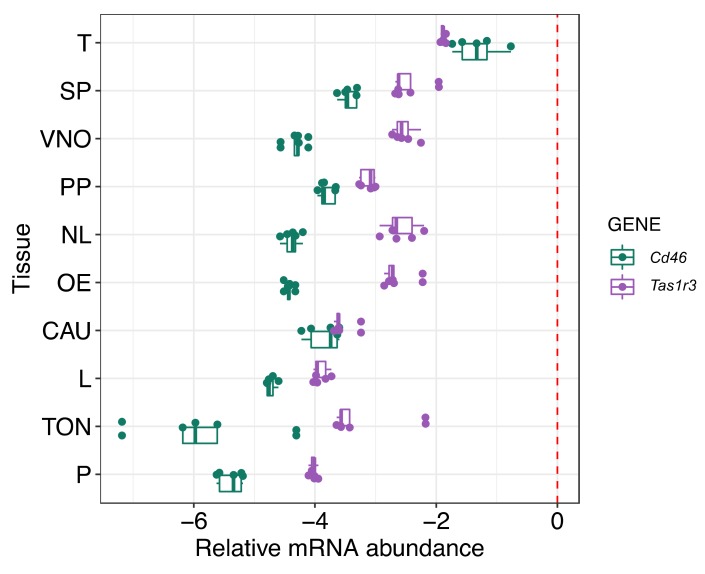
The expression of *Tas1r3* and *Cd46* is highest in testicles as revealed by qPCR analysis of mRNA across 10 mouse tissues. Prostate (P), tongue (TON), liver (L), cauda epididymis (CAU), olfactory epithelia (OE), lymph tissue (NL), nasal-associated major preputial gland (PP), Vomeronasal organ (VNO), spleen (SP) and testis (T). Normalized to *Gapdh* (dashed red line), *n*_male_ = 5.

A total of 10 tissues were selected for mRNA screening to assess the relative importance of given genes in each tissue based on the expression differences (Figure 1). Relative abundance of *Tas1r3* mRNA was highest in testis and the level of abundance when compared to other tissues was closest to the level of *Gapdh* (housekeeping gene) delimited by the red dashed line in Figure 1. Interestingly, *Tas1r3* expression in testis was ~two-fold higher than that in the tongue where this receptor was originally detected. This expression pattern of *Tas1r3* resembles the one of *Cd46* [27], which was used as a positive control due to its abundant expression in testes and its well described role in fertilization of mammals especially rodents [7,8,28,29]. The result of mRNA gene expression (Figure 1) suggests that the mTAS1R3 receptor, similar to CD46, is expressed in testes including male germ cells; therefore, it could be predicted to be involved in sperm-related fertilization strategies.

### 2.2. Localization of mTAS1R3 During Sperm Maturation

**Figure 2:**
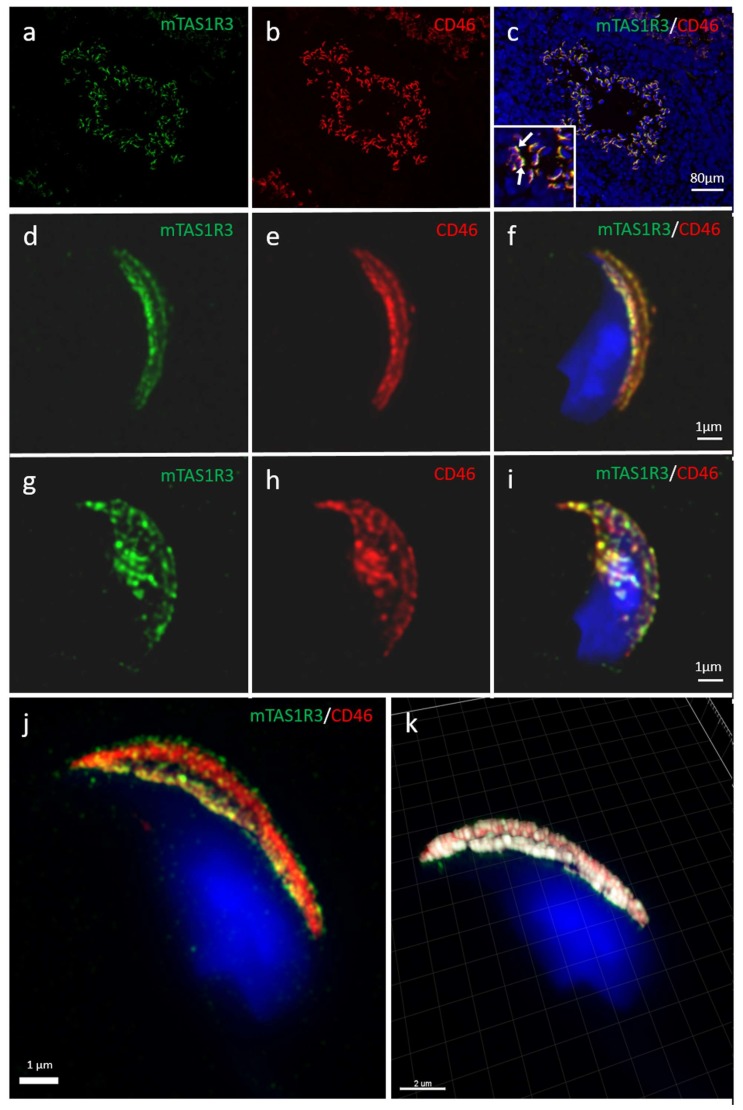
Localization of mTAS1R3 (green) and CD46 (red) in mouse sperm revealed by Confocal Microscopy and Structure Illumination Microscopy (SIM). During spermiogenesis (**a**) mTAS1R3 (green) and (**b**) CD46 (red) are localized in elongated and late spermatids with a formed acrosome (**c**) both proteins colocalize (yellow); for details, see enlarged area and white arrows pointing to the spermatids. (**d**) In epididymal sperm, mTAS1R3 (green) is present in the apical acrosome specifically in the acrosomal membranes and corresponds to (**e**) CD46 (red) localization. (**f**) The colocalization of both mTAS1R3 and CD46 pattern (yellow) is shown in acrosomal membranes defining the intact acrosome overlaying the nucleus (blue). (**g**) During the acrosome reaction mTAS1R3 (green) relocates into the equatorial segment, as well as (**h**) CD46 (red) and (**i**) their colocalization (yellow) is shown with nucleus (blue) overlay. (**j**) SIM imaging shows precise localization of mTAS1R3 (green) in the acrosomal membranes. (**k**) Huygens software was used for better visualization of mutual position of mTAS1R3 (green) and CD46 (red) in acrosomal cap area. White color shows the place of colocalization with CD46. CD46 was used as a marker of the acrosomal membranes. Scale bars represent (**a**–**c**) 80 μm, (**d**–**j**) 1 μm, (**k**) 2 μm.

A previously-reported taste receptor, mTAS1R3, found in the sperm head [10], was identified as a potential protein involved in the chemical communication of gametes, particularly in sperm chemotaxis to the oocyte due to its ability to bind to the *L-glutamate* present in the female reproductive tract [23]. We aimed to identify in detail mTAS1R3 sperm head localization and target the receptor behavior during sperm maturation with a special focus on its compartmental localization during membrane rearrangements during the acrosome reaction. For mTAS1R3 sperm detection, super-resolution capturing was used in parallel to indirect immunofluorescence labelling of testicular sections. To determine the precise localization of mTAS1R3 during mouse sperm maturation, we used double immunostaining with CD46 protein (Figure 2a–c) as a marker of acrosomal membranes in intact mouse sperm [28]. During sperm development in testis, both mTAS1R3 and CD46 immunofluorescent signals were detectable in elongating and late spermatids when the acrosome is formed (Figure 2a–c, and Figure 2c enlarged area with white arrows). These findings were confirmed using *testes* of our transgenic mouse line C57BL/6N^acr3-EGFP^, expressing green fluorescent protein in the acrosome of spermatids and sperm [30] (Figure S1). Using dual color immunofluorescent staining visualized by confocal microscopy, we detected a very similar localization of mTAS1R3 and CD46 in the acrosomal cap area in freshly released acrosome-intact sperm (Figure 2d–f). Moreover, both proteins showed similar behavior in sperm undergoing AR, where mTAS1R3 (Figure 2, labelled green) as well as CD46 (Figure 2, labelled red) relocated from the acrosomal cap area (Figure 2d–f) to the equatorial segment (Figure 2g–i), which exposes the protein surface after AR (Figure 2g–i). We used Structured illumination microscopy (SIM), which provided a more accurate resolution of images than confocal microscopy, for the determination of the precise localization of mTAS1R3 within the acrosomal cap area of the intact sperm head (Figure 2j). Additionally, Huygens software was used to create a colocalization map of mTAS1R3 and CD46 based on Pearson’s correlation coefficient for the visualization of the colocalization area of both proteins and to improve the identification of individual membranes within the acrosomal cap with expression of mTAS1R3 (Figure 2k). These results showed that mTAS1R3 is present in both the inner and outer acrosomal membranes. The application of secondary antibodies without presence of primary antibodies confirm the specificity of the signal for mTAS1R3 and CD46 (Figure S2).

The detailed monitoring of the mTAS1R3 receptor provided an understanding of the position of mTAS1R3 during the final stages of sperm maturation, when in vivo sperm is in close proximity to the egg and its surroundings (*zona pellucida* and *cumulus oophorus* cells). At this stage, the sperm–egg short distance chemical interaction is predicted to take place and the mTAS1R3 receptor is the surface exposed on the sperm head.

### 2.3. Sperm Chemotactic Responsiveness to L-glutamate

**Figure 3:**
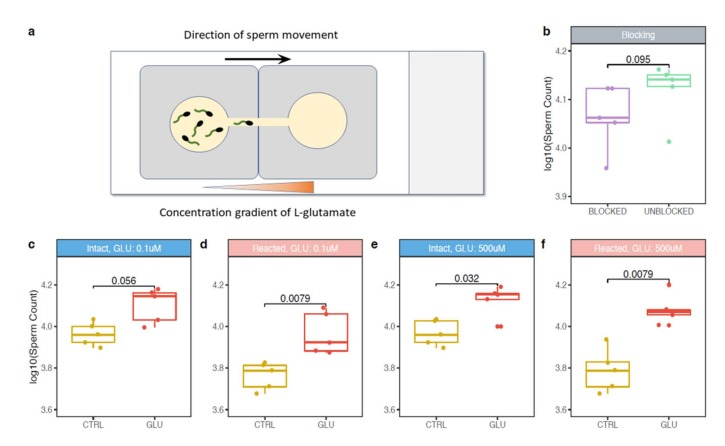
Sperm chemotactic response to *L-glutamate*. (**a**) Special chemotactic chamber was developed for assessing sperm attraction to *L-glutamate*. Wells were filled with M2 medium containing spermatozoa (left) and with *L-glutamate* (right) and connected through the bridge. The black arrow represents the direction of sperm movement, whereas the orange triangle represents the concentration gradient of L-glutamate. SecureSeal imaging spacers are in grey. (**b**) Comparison of chemotactic response of acrosome-reacted sperm to *L-glutamate* in the presence or absence of a specific goat polyclonal anti-mouse mTAS1R3 antibody, n_male_ = 5. The response to (**c,e**) acrosome-intact and (**d,f**) acrosome-reacted sperm to *L-glutamate* was analyzed in (**c,d**) 0.1 μM and (**e,f**) 500 μM concentrations.

*L-glutamate* is present in the female genital tract [23] and may potentially function as a chemoattractant. Based on our reported evidence that the mTAS1R3 is being localized in the intact acrosome of epididymal sperm and then relocates during the acrosome reaction when it becomes surface exposed, we decided to examine a possible chemotactic attraction of both intact and acrosome-reacted sperm to *L-glutamate*. Chemotaxis of epididymal acrosome-intact and acrosome-reacted sperm to two different concentrations of *L-glutamate* were investigated (Figure 3). We selected two experimental concentrations of 500 μM and 0.1 μM for *L-glutamate* in accordance with relevant available literature [19,20,21], where 500 μM was previously used to study a role of glutamate as a neurotransmitter in the central nervous system [31,32], and a concentration of 0.1 μM was used for analysis of *L-glutamate* uptake by sperm [33]. The authors [33] demonstrated a presence of *N*-methyl-D-aspartate (NMDA) glutamate receptor NR2B and sodium-dependent glutamate transporter GLT1 in mouse and human sperm and showed that mouse sperm possessed the glutamate uptake property in similar way as brain did. Due to the fact that the acrosome vesicle exocytosis displays similar physiology with the active zone of the presynaptic terminal in neurons where neurotransmitter vesicle fusion occurs, and thus may be termed “acrosomal synapse” [34], the research from the field of neuroscience is relevant to sperm reproductive physiology.

In our research, we targeted the short distance sperm–egg chemical communication when sperm had reached a fully mature stage, had completed capacitation and was undergoing or had successfully completed AR. For assessing a predicted sperm targeted attraction to *L-glutamate*, two modified SecureSeal imaging spacers were used (Figure 3a, see Methods for details). We applied the Shapiro-Wilk normality test, which revealed that the data does not deviate from normality (*p* = 0.219); however, the sample size was not sufficient to use parametric tests. Thus, we applied the Wilcoxon signed rank test to detect the role of antibody blocking (Figure 3b) and the influence of *L-glutamate* on the sperm swimming capacity to reach the highest concentrations (Figure 3c–f). In lower concentrations of *L-glutamate* (0.1 μM), there was only a trend (*p* = 0.056) for acrosome-intact sperm being more attracted to *L-glutamate* (Figure 3c). In comparison, the acrosome-reacted sperm showed significant attraction to 0.1 μM *L-glutamate* (0.0079) (Figure 3d). In 500 μM *L-glutamate* concentration, the acrosome-intact sperm were also significantly attracted to *L-glutamate* (*p* = 0.032) (Figure 3e), however, this effect was strongly profound and more significant for the acrosome-reacted sperm (*p* = 0.0079) (Figure 3f). To conclude our findings, the acrosome-reacted sperm had better capacity to reach the place with higher concentrations of *L-glutamate* than acrosome-intact sperm.

Furthermore, we aimed to corroborate these findings with another experiment where acrosome-reacted sperm were incubated with a specific goat polyclonal anti-mouse mTAS1R3 antibody (ABIN571574, antibodies-online GmbH) to detect potential changes in sperm responsiveness to *L-glutamate* and selected the lower concentration (0.1 μM), to which the response of acrosome-reacted sperm compares to acrosome-intact sperm was significantly different. The Wilcoxon signed rank test revealed that the differences between the control untreated sperm and sperm incubated with the anti-mTAS1R3 antibody with a blocked mTAS1R3 receptor were not significant (*p* = 0.095); however, there was an obvious trend that after the antibody blocking the response of acrosome-reacted sperm to *L-glutamate* was strongly decreased. (Figure 3b).

Because we are under the impression that mTAS1R3 plays a role in short distance chemotaxis of sperm in close proximity to cumulus-oocytes complex (COC), we investigated if the exposition of acrosome-reacted sperm to anti-mouse mTAS1R3 antibody influences their chemotactic response to COC. Preliminary data were collected from in vitro experiments in the presence of COC. The mouse eggs with *zona pellucida* and *cumulus oophorus* cells were placed into a chamber (Figure 3a) and a chemotaxis of anti-mouse mTAS1R3 antibody-exposed acrosome-reacted sperm was compared to acrosome-reacted sperm without the presence of the antibody. There was a significantly lower number of the acrosome-reacted sperm in the presence of the antibody reaching the egg chamber and it would be interesting to explore these promising data in further detail.

## 3. Discussion

The evidence that mTAS1 receptors are present in the mouse sperm head [10] opens a question about their function. With the identification of olfactory receptors on sperm, a correlation was made between olfaction and sperm orientation in the female reproductive tract [25,35,36] and it is plausible that the action of mTAS1 receptors on sperm might be a mediation of sperm guidance towards the oocyte through the process called chemotaxis. This theory is supported by the fact that binding ligands of TAS1 receptors, known from taste, are present in female genital tract fluid, which represent a natural source of chemoattractants [23]. Additionally, TAS1 belongs to a group of G-coupled receptors, and during the activation of signaling pathways controlling chemotaxis, the modulation of adenylate cyclase and phospholipase C by receptors coupled with G-protein occurs [37]. However, to the best of our knowledge, there is no available research testing this hypothesis.

In order to address this gap, we focused on mTAS1R3, a receptor present on male gametes, to investigate its potential role in sperm chemotaxis. Although the action of taste receptors was originally described for the tongue, they are also present in many other tissues; we have shown *Tas1r3* expression in 10 different types of mouse tissue in addition to the tongue. Interestingly, it was discovered that the mRNA expression level was highest in the testis (Figure 1). This result indicated the importance of mTAS1R3 in reproduction and mainly in male fertility, which is supported by the evidence that TAS1 receptors are involved in spermiogenesis or the post-testicular maturation of sperm [5,10].

TAS1R3 belongs to the TAS1R protein family, which contains three members of taste buds, but the expression of only two of them, TAS1R3 and TAS1R1, whose presence has been proven in mouse and human sperm [10]. In the oral cavity, TAS1R1 and TAS1R3 create a heterodimer that is responsible for the recognition of the umami taste, but their dimerization has not yet been confirmed in sperm. Nevertheless, the ligand of the umami taste receptor differs between humans and mice. While in the case of humans, *L-glutamate* is exclusively a ligand of the umami receptor, in mice, the receptor is activated by all L-amino acids [20]. However, it is generally presumed that chemoattractants are universal rather than species-specific in mammals [1]. For this reason, *L-glutamate* seems to be a suitable candidate molecule serving as a chemoattractant, and previously-described evidence about the accumulation of glutamate in pre-ovulation follicular fluid [38], as well as the fact that glutamate was found to be present in the reproductive tract [23], support this theory. The metabolomic profile of bovine cumulus cells during oocyte maturation in vitro was investigated, and it was shown that glutamate is one of the COC products and its concentration increases in maturation medium with time [39]. These findings correlate with results from a human study [38], which showed that the concentration of glutamate in pre-ovulatory follicles is 70.0 ± 3.80 µM. This concentration is three times higher than in plasma (23.18 ± 2.20 µM) and it suggests that there exists an accumulation of glutamate in follicular fluid. On the other hand, in mouse [23], the follicular fluid glutamate concentration (0.277 ± 0.022 mM) was similar to the one in plasma (0.322 ± 0.037 mM); however, the in vitro maturation experiment was not performed and the actual COC glutamate production after the ovulation was not measured. Therefore, a glutamate concentration gradient in COC surroundings can exist. Moreover, it could be the gradient that is required for sperm chemotaxis. In our experimental set up, two widely different glutamate concentrations were used.

Although the presence of mTAS1R3 in the acrosomal cap area was previously described [10], its precise localization in the individual membranes remained unclear. We used dual immunofluorescent staining of mTAS1R3 and CD46 as a marker of acrosomal membranes [28] and used super resolution microscopy (SIM) to better distinguish the individual membranes. Based on this method, we show that mTAS1R3 is localized in the outer and inner acrosomal membranes in the acrosome-intact sperm and that it is not surface-exposed during sperm capacitation, which takes place when sperm pass through the oviduct (Figure 2d–j). The acrosomal vesicle and its status is a crucial indicator of the sperm quality and maturation state. When the acrosome is released, the sperm is in close proximity to the egg; however, in mice, this happens before the *zona pellucida* is reached [9]. This physiological phenomenon of spontaneous AR was also described and significantly increased in sperm of *Tas^−/^*^−^ [10] as well *Cd46^−/−^* [29] mice. For this reason, monitoring the protein localization prior to and after the acrosome reaction provides valuable clues about this protein’s function. As a next logical step, we, therefore, focused on the receptor behavior during the AR. We provide evidence that after the AR, mTAS1R3 is relocated into the equatorial segment overlaying the apical part of the sperm nucleus (Figure 2g–k). This segment is crucial in first communication between sperm and egg and it is defined by the inner acrosomal membrane and remaining plasma membrane. This occurs only after the AR, when mTAS1R3 becomes surface-exposed to ligands in the outer surroundings of the female reproductive tract, where *L-glutamate* is also present. Many proteins are released from the sperm during AR, either as soluble compounds or in membranes of hybrid vesicles formed by the plasma and outer acrosome membranes. It is not by accident that a protein is relocalized during the AR event; therefore, we progressed towards conducting chemotactic experiments with both epididymal acrosome-intact and acrosome-reacted spermatozoa. In conjunction with previous findings, we detected that the response of acrosome-reacted sperm to *L-glutamate* stimuli is significant in contrary to acrosome-intact sperm (Figure 3c,d). However, the reason why sperm head acrosomal status should be important for the chemotactic response is still unclear. In the case of progesterone, a known chemoattractant of mammalian sperm, it was reported that mouse spermatozoa require an intact acrosome to display the chemotactic response [40]. This is probably because the presence of the progesterone receptor was identified in the plasma membrane of the sperm overlaying the acrosomal area [41]. On the other hand, the observation that mouse sperm undergoes AR while passing through the upper isthmus of the oviduct [12] suggested that there were probably other molecules that guide the acrosome-reacted sperm through the ampulla. We could hypothesize that if mouse sperm undergo a physiologically known premature spontaneous reaction prior to reaching the *zona pellucida* of the egg but with close enough proximity, the communication between mTAS1R3 and *L-glutamate* can provide a particularly effective “short distance” chemotactic guidance mechanism. The spontaneous AR could also be one of the reasons why we observed some chemotaxis in sperm without the AR induction. Even though we call these sperm acrosome-intact, about 15% of them must have undergone the physiological spontaneous AR when protein dynamics, and their fertilization potential remains the same as when the AR is induced by other stimuli [42]. In addition, the presence of mTAS1R1 and mTAS1R3 [10], as well as glutamate specific NR2B receptors [33], were also shown on the sperm tail. The NR2B receptors belong to the family of NMDR receptors, which were shown to mediate the AR in newt sperm, and when blocked, a significant decrease of both induced and spontaneous AR as well as motility was observed [43].

To challenge this hypothesis, we used a specific anti-mTAS1R3 antibody to block the interaction between mTAS1R3 and *L-glutamate*. The acrosome-reacted sperm in the presence of anti-mTAS1R3 antibody showed a lower chemotactic response to *L-glutamate* compared to the control group without antibody blocking and even though the differences were not statistically significant, there was an evident decree of chemotactic response to *L-glutamate* in the number of sperm exposed to the antibody (Figure 3b). The fact that differences between both groups were not significant could be explained in two different ways or by a combination of both. Firstly, the antibody did not cover the whole ligand-binding site and the receptor was blocked only partially. Additionally, in case of a predicted formation of a heterodimer with mTAS1R1, the binding site might be better targeted if both monomers were blocked. Targeting mTAS1R1 is also relevant, as it has been shown to participate in the regulation of Ca^2+^ and cAMP concentrations in sperm [10]; therefore, mTAS1R3 might have a similar role. This seems plausible, since TAS1R3 and TAS1R1 are expected to form a heterodimer in sperm similarly to their already confirmed dimerization in the tongue tissue [10]. Secondly, mTAS1R3 is not the only sperm receptor binding *L-glutamate* that participates in sperm chemotactic response. This explanation is supported by the fact that the presence of metabotropic glutamate receptors (mGluRs) were described in testis and in mature sperm [44]. As well as mTAS1R3, mGluRs belong to the G-protein coupled receptor family and trigger cAMP/Ca^2+^ signaling pathway [45]. Thanks to their localization in the midpiece and sperm flagellum, they are believed to be involved in sperm motility [33]. For this reason, it could be that mGluRs might participate in the sperm chemotactic response to *L-glutamate* in conjunction with mTAS1R3. In addition to the above discussed points, it will be beneficial to address in detail the egg-side of the story and build on our preliminary findings.

## 4. Material and Methods

### 4.1. Animals

Inbred C57BL/6J mice and reporter transgenic male mice (C57BL/6J^acr3-EGFP^) expressing EGFP in the acrosome of developing spermatids and sperm were housed in a breeding colony of the Laboratory of Reproduction, IMG animal facilities, Institute of Molecular Genetics of Czech Academy of Science, and food and water were supplied ad libitum. C57BL/6J^acr3-EGFP^ were generated according to [30] in the Transgenic Unit of the Czech Center for Phenogenomics at the Institute of Molecular Genetics CAS, and are the property of the Laboratory of Reproductive Biology, Institute of Biotechnology of the Czech Academy of Sciences. The male mice used for all experiments were healthy, 10–12 weeks old, with no sign of stress or discomfort. The super-ovulated C57BL/6 females were 23–26 days old. All the animal procedures and experimental protocols were approved by the AnimalWelfare Committee of the Czech Academy of Sciences (Animal Ethics Number 66866/2015-MZE-17214, 18 December 2015).

### 4.2. qPCR

Total RNA was isolated from ten different tissues (vomeronasal organ (VNO), main olfactory epithelium (OE), nasal-associated lymphoid tissue (NL), preputial gland (PP), prostate (P), cauda epididymis (CAU), testes (T), spleen (SP), liver (L) and tongue (TON)) of C57BL/6 males, n_male_ = 5. RNA isolation was performed using TriReagent solution (Sigma-Aldrich) according to standard chloroform/isopropanol protocol (https://www.sigmaaldrich.com/technical-documents/protocols/biology/tri-reagent.html) and followed by DNase protocol (Fermentas, Waltham, MA, USA) to avoid potential DNA contamination. cDNA was synthetized with RevertAid M-MulV Reverse Transcriptase using Oligo (dT) primers (First-strand cDNA synthesis kit, Fermentas). Quantitative PCR (qPCR) was performed on LightCycler480 using SYBR Green I Master chemistry according to manufacturer’s protocol (Roche Applied Sciences, Penzberg, Germany). Primers were designed as intron spanning assay and the sequences are as follows: GapdhF-5′ ATG GTG AAG GTC GGT GTG A 3′, GapdhR-5′ AAT TCT CCA CTTTGC CAC TGC 3′, Cd46F-5′ AGC CCT CCG GAG TGT AAA GT 3′, Cd46R-5′ ACA TCA CTG TTG ATT GAT AGG AAA AT 3′, Tas1r3F-5′ ACA GGT TCT CAC CCC TTG G 3′, Tas1r3R-5′ TCT CCT CCA CAG CCA TCT TC 3′. Each sample ran as triplicate; non-template and non-RT reactions were used as controls. Conditions of qPCR amplification were optimized to: initial denaturation at 95 °C for 10 min, followed by 35 cycles consisting of denaturation at 95 °C for 10 s, annealing at 56 °C for 10 s and extension step at 75 °C for 10 s. After the cycles were completed, we set up the melting analysis to check the specificity of the amplification. The Cp value was obtained for each sample and triplicate variation was controlled to not exceed standard deviation 0.5 Cp. Average values of triplicates were than used to calculate relative expression according to the housekeeping gene (Gapdh). Because the expression of target genes (*Cd46, Tas1r3*) was in some tissues much lower than the expression of the housekeeping gene, we used logarithmic scale (Log10) for data visualization in the graph (Figure 1).

### 4.3. Mouse Testes Cryosection Preparation

Isolated testes of inbred C57BL/6J and C57BL/6J^acr3-EGFP^ mice, *n*_male_ = 3 were placed in Tissue Tek OCT compound, 4oz (4583, Electron Microscopy Sciences, Hatfield, PA, USA) and frozen in −80 °C Using cryostat (Leica CM1950, Wetzlar, Germany), testes were sectioned into 6 μm slices, which were fixed in ice-cold methanol-acetone for 10 min. Sections were washed in PBS for 5 min and kept at −20 °C before further usage.

### 4.4. Sperm Sample Collection

Isolated distal regions of *caudae epididymidae* of inbred C57BL/6J and C57BL/6J^acr3-EGFP^ mice, *n*_male_ = 6 were placed into Petri dishes, each with two 200 μL droplets of M2 medium covered with high viscous paraffin oil (P14501, Carl Roth, Germany) pre-tempered at 37 °C under 5% CO_2_, and left in the incubator (set on 37 °C, 5% CO_2_) for 10 min for sperm to be released.

### 4.5. Sperm Capacitation and Acrosome Reaction

The above-described released spermatozoa in amount of 5 μL were transferred to another Petri dishes prepared as described in 4.4., with the only difference of 100 μL M2 medium droplets. After 90 min of capacitation, the acrosome reaction was induced by Calcium Ionophore (A23187, Sigma-Aldrich, Prague, Czech Republic) at a final concentration of 5 μM for further 90 min as described previously [28]. All along, Petri dishes were kept in the incubator set on conditions as described in 4.4. The acrosome-reacted sperm were used for immunofluorescent analysis *n*_male_ = 6, or placed in fresh M2 medium to recover motility and to be used for chemotactic responsiveness experiment, *n*_male_ = 5, please see methods 4.7.

### 4.6. Immunofluorescent Detection of mTAS1R3 with Confocal and Structured illumination Microscopy (SIM)

Epididymal acrosome-intact and acrosome-reacted sperm (*n*_male_ = 5) and testis (*n*_male_ = 3) cryosections were used for confocal microscopy and SIM. Experiments were repeated six times (*n*_experiment_ = 6) from each male and only representative results are shown. Sperm were collected from Petri dishes, washed twice in PBS and smeared onto a glass slide. For SIM, sperm samples were always prepared onto high-precision cover glasses (thickness No. 1.5 H, 170 ± 5 μm, Marienfeld, Germany). Air-dried smears were fixed with ice-cold methanol-acetone, 5 min, −20 °C. After washing in PBS, sperm were treated with 5% BSA in PBS for 45 min. Testes cryosections were hydrated in PBS for 5 min and also blocked in 5% BSA. Glass slides were incubated over night at 4 °C with primary antibody goat polyclonal anti-mouse mTAS1R3 (ABIN571574, antibodies-online GmbH, Aachen, Germany) 1:100, for cryosections 1:200 and rat monoclonal anti-mouse CD46 MM10 antibody (HM-1118, HycultBiotech, Uden, The Netherlands) 1:50, for cryosections 1:100 diluted in PBS. After washing in PBS, secondary antibodies Alexa Fluor 488 donkey anti-goat IgG (H+L), and Alexa Fluor 568 goat anti-rat IgG (H+L) (Molecular Probes, Eugene, OR, USA), all diluted in PBS 1:300, for cryosections 1:500 were applied for 1 h. In case of dual staining, both primary and secondary antibodies were applied together. For SIM, after the application of the primary and secondary antibodies, sperm were incubated for 5 min with DAPI (0.85 μg/mL, Thermo Scientific, Waltham, MA, USA). Samples were washed 3× in PBS and at the end, sperm were washed 1× in distilled water and air-dried. Dry samples were covered with 90% glycerol with 5% anti-fade *N*-propyl gallate (Sigma-Aldrich) for SIM and Vectashield mounting medium with DAPI (Vector Laboratories, Burlingame, CA, USA) for confocal microscopy. Multi-color SIM super-resolution images were obtained by Zeiss Elyra PS.1 inverted microscope at the Laboratory of confocal and fluorescent microscopy of the Faculty of Science (Charles University, Prague, Czech Republic). Fluorescent images were taken with an Olympus IX81 fluorescent microscope or high-end confocal microscope Carl Zeiss LSM 880 NLO at Imaging Methods Core Facility at BIOCEV (Vestec, Czech Republic) and processed in an open-source software Fiji [46]. Huygens Professional version 19.04 (Scientific Volume Imaging, Hilversum, The Netherlands, Available online: http://svi.nl) was used for deconvolution and visualization of mutual position of mTAS1R3 and CD46 based on surface rendering of the colocalization analysis. A colocalization analyzer computed a Pearson’s correlation coefficient and created a three-dimensional (3D) colocalization map. Corresponding negative controls were carried out similarly to other glass slides with the difference that PBS was used instead of primary antibodies.

### 4.7. Sperm Chemotactic Responsiveness to L-glutamate

For this experiment, epididymal acrosome-intact and acrosome-reacted sperm of C57BL/6 males, n_male_ = 5, were prepared as described above in 4.4. and a glass slide with two SecureSeal imaging spacers (Sigma-Aldrich) were used. In both imaging spacers, a thin line (approximately 1 mm) between the well and the edge was cut out and imaging spacers were adhered on the glass slide with cut lines next to each other forming a bridge connecting the two wells. M2 media with two different concentrations (500 μM and 0.1 μM) of *L-glutamate* (G-1501, Sigma-Aldrich) were prepared. Wells were filled with 100 μL of M2 medium containing 4 μL released spermatozoa and with 100 μL of *L-glutamate*, in the case of negative control with 100 μL of pure M2 medium without glutamate. Wells were then carefully connected through the bridge by a pipette tip in the direction from glutamate drop to avoid a passive drag of sperm to glutamate. After 5 min, the drop with glutamate (or with pure M2 medium) was collected and sperm counted in Bürker chamber (3–5 main squares were analyzed). Every measurement was repeated three times. To compare the counted numbers from each individual, counts were recounted according to the initial concentration of the used spermatozoa. Throughout the experiment, the sperm and glass slide were kept in the incubator. We used M2 medium and glutamate were prewarmed. Sperm viability and motility was checked at the beginning and at the end of each experiment under the light microscope.

### 4.8. Sperm Chemotactic Response to L-glutamate in Presence of Antibody

To address the potential involvement of the mTAS1R3 receptor in mediation of sperm chemotactic response, a specific goat polyclonal anti-mouse mTAS1R3 antibody (ABIN571574, antibodies-online GmbH) was added to epididymal capacitated spermatozoa from C57BL/6 mice, n_male_ = 5 (in total concentration 1:100). Sperm further underwent the acrosome reaction under the same conditions as previously described in 4.5. As a control group, epididymal acrosome-reacted sperm that were not incubated with the antibody were used. The concentration of *L-glutamate* in M2 medium was 0.1 μM. The rest of the procedure was performed in the same way as described in 4.7.

### 4.9. Sperm Chemotactic Response COC in Presence of Antibody

To answer the question if mTAS1R3 plays role in short distance chemotactic response in close proximity to COC, we repeated experiment described above in paragraph 4.8 with the following difference: instead of a drop of M2 medium with *L-glutamate*, the COCs were placed into the drop of M2. The eggs with *cumulus oophorus* cell were harvested from the hyper-stimulated female mice by following protocol. Female mice were hormonally stimulated with 5UI PGMS—Pregnant Mare’s Serum Gonadotropine (Folligon, Intervet International B.V., Boxmeer, The Netherlands) at 3 p.m. (eighth hour of light cycle) on the first day of protocol. 5UI of hCG—human Chorionic Gonadotropin (CG10, Sigma-Aldrich, St. Louis, MI, USA) were applied to mice at 1 p.m. third day of protocol (46^th^ hour after using PGMS). After 12 h, females started ovulating. At 9 a.m. on the fourth day of protocol, female mice were sacrificed by cervical dislocation and both ampullas of fallopian tube were isolated and placed in preheated M2 medium (M7167, Sigma-Aldrich, St. Louis, MI, USA). COC was released into M2 medium by ampulla tearing.

## 5. Conclusions

The localization of mTAS1R3 suggests that this receptor may participate in yet to be defined signal sensing in the acrosome-reacted sperm. The chemotaxis experiments support this conclusion, although they also indicate that there may be other receptors reacting to glutamate because the acrosome-intact sperm also showed the chemotaxis response and anti-mTAS1R3 antibody did not block the chemotaxis completely.

## Supplementary Materials

Supplementary materials can be found at https://www.mdpi.com/1422-0067/21/7/2651/s1. Figure S1: Localization of mTAS1R3 in mouse testicular tissue of transgenic mouse line C57BL/6N^acr3-EGFP^, expressing green fluorescent protein in the acrosome of spermatids and sperm. Figure S2: Negative controls, the reaction of secondary antibodies with testicular tissue and epididymal spermatozoa.
