# Supplementary material for: The Role of Taste Receptor mTAS1R3 in Chemical Communication of Gametes
Source: Int J Mol Sci. 2020 Apr 10;21(7):2651. doi: 10.3390/ijms21072651 (PMC7177404; doi:10.3390/ijms21072651)
Supplement: Supplementary file 1 [file ijms-21-02651-s001.pdf]

The Role of Taste Receptor mTAS1R3 in Chemical Communication of Gametes

Michaela Frolikova [1], Tereza Otcenaskova [1,2], Eliska Valasková [1], Pavla Postlerova [1,3], Romana Stopkova [2], Pavel Stopka [2] and Katerina Komrskova [1,2,*]

[1] Laboratory of Reproductive Biology, Institute of Biotechnology of the Czech Academy of Sciences, BIOCEV, Prumyslova 595, 252 50 Vestec, Czech Republic
[2] Department of Zoology, Faculty of Science, Charles University, BIOCEV, Vinicna 7, 128 44 Prague 2, Czech Republic
[3] Department of Veterinary Sciences, Faculty of Agrobiology, Food and Natural Resources, University of Life Sciences Prague, Kamycka 129, 165 00 Prague 6, Czech Republic
* Correspondence: katerina.komrskova@ibt.cas.cz; Tel.: +420-325-873-799

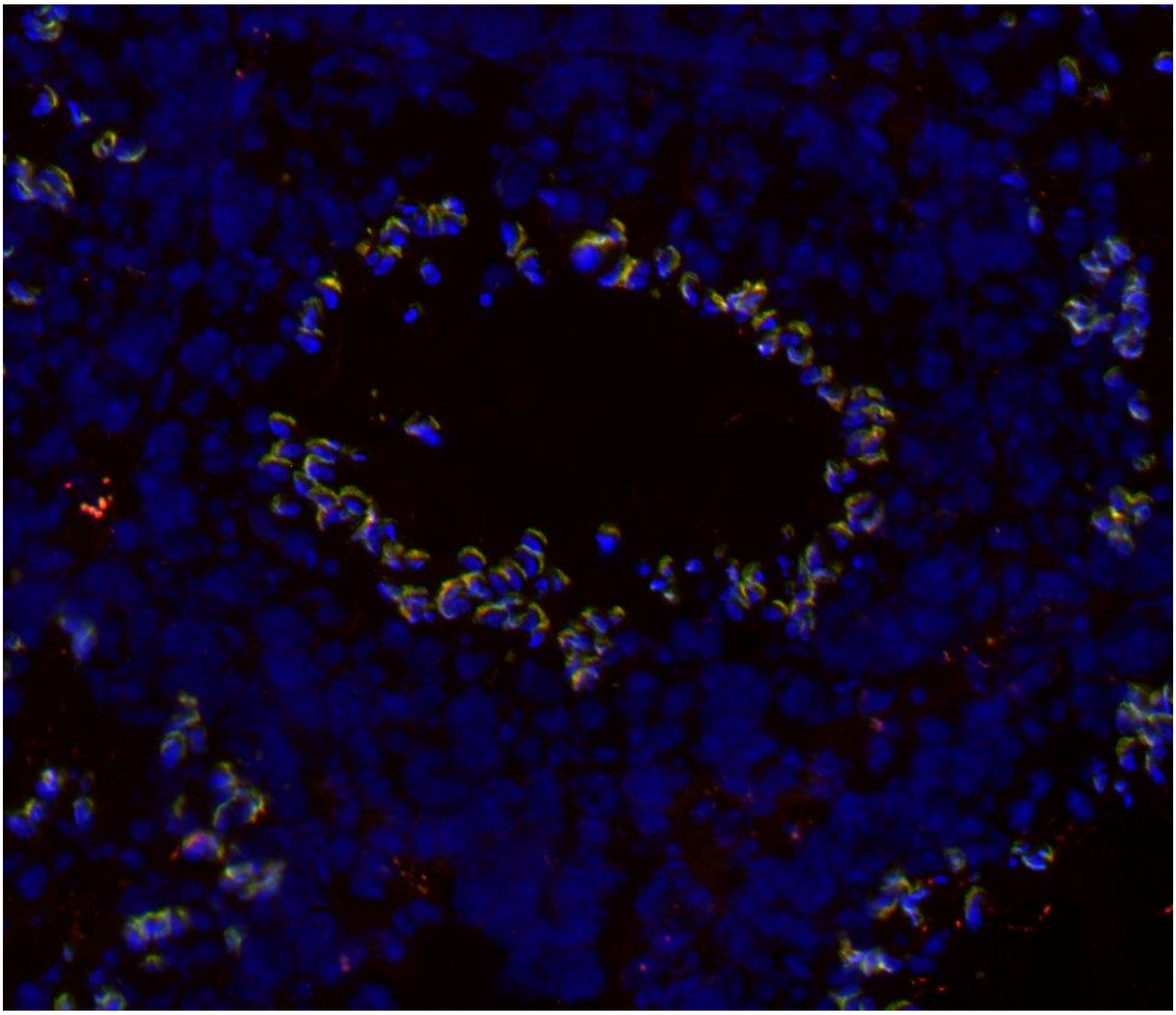

Figure S1 Localization of mTAS1R3 (green) in mouse testicular tissue of transgenic mouse line C57BL/6Nacr3-EGFP, expressing green fluorescent protein in the acrosome of spermatids and sperm. During spermiogenesis (a) mTAS1R3 (red) and (b) EGFP (green) are localized in spermatids with a formed acrosome where (c) both proteins colocalize (yellow).

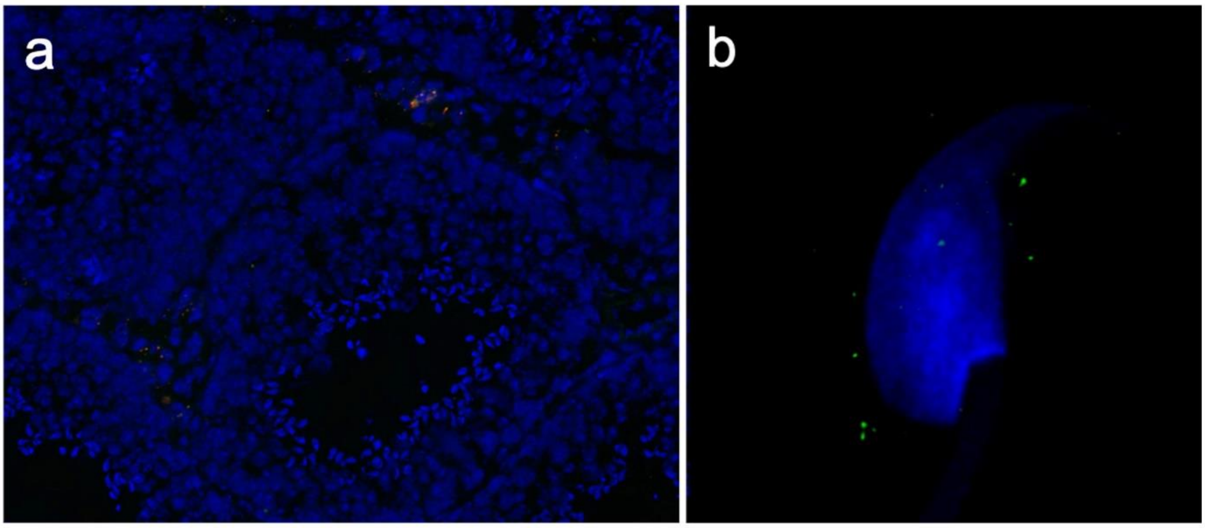

Figure S2: Negative controls, the reaction of secondary antibodies with testicular tissue and epididymal spermatozoa. No specific signal was detected after application of secondary antibodies Alexa Fluor 488 donkey anti-goat IgG (H+L), and Alexa Fluor 568 goat anti-rat IgG (H+L) without primary antibodies on **(a)** testicular tissue and **(b)** epididymal spermatozoa.
